# Environmental Remediation with Functional Aerogels and Xerogels

**DOI:** 10.1002/gch2.202000013

**Published:** 2020-06-17

**Authors:** Brian J. Riley, Saehwa Chong

**Affiliations:** ^1^ Pacific Northwest National Laboratory 902 Battelle Blvd Richland WA 99352 USA

**Keywords:** aerogels, environmental remediation, silver functionalization, xerogels

## Abstract

Several different types of aerogels and xerogels are demonstrated as effective sorbents for the capture and/or immobilization of radionuclides and other contaminants in gaseous form [e.g., Hg_(g)_, I_2(g)_, Xe, Kr] as well as ionic form (e.g., Cd^2+^, Ce^4+^, Cs^+^, Cu^2+^, Fe^2+^, Hg^2+^, I^−^, IO_3_^−^, Kr, Pb^2+^, Rb^+^, Sr^2+^, ^99^Tc^7+^, U^6+^, Zn^2+^). These sorbents have unique properties, which include high specific surface areas, high pore volumes, a range of pore sizes, and functionalities that provide methods for binding radionuclides and other contaminants, generally through physisorption, chemisorption, or a combination thereof. This combination of properties and functionalities makes these types of materials ideal for use as sorbents for capturing radionuclides. The primary base materials that will be discussed in this paper include Ag^0^‐functionalized silica aerogels, Ag^+^‐impregnated aluminosilicate aerogels, Ag^0^‐functionalized aluminosilicate aerogels, metal‐impregnated (non‐Ag) aluminosilicate aerogels and xerogels, sulfide‐based aerogels, and carbon‐based aerogel composites. For the capture of I_2(g)_, the materials reported herein show some of the highest iodine loadings ever reported for inorganic sorbents. For the capture of ionic species, these materials also show promise as next‐generation materials for active radionuclide remediation. This progress report describes materials fabrication, general properties, and environmental remediation applications.

## Graphical abstract

Functionalized aerogels and xerogels are demonstrated for use in the removal of contaminants from aqueous and gaseous streams, including species such as halides, noble gases, actinides, lanthanides, alkalis, and alkaline earths that could pose environmental and health concerns if not captured and contained properly. This paper provides a progress report of the current state of the art in this area.

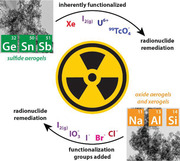

## 1 Introduction

Porous sorbents including aerogels, xerogels, metal–organic frameworks (MOFs), and zeolites have received a lot of attention over the past several decades for environmental remediation applications. These sorbents have a variety of uses including the purification of gaseous and aqueous streams through chemisorption, physisorption, ion exchange, molecular sieving, and combinations thereof. All these materials have high specific surface areas (*SSA*), which are measured in terms of surface area per mass of material (i.e., m^2^ g^−1^), with values ranging from a few hundred m^2^ g^−1^ (e.g., zeolites) to several thousand m^2^ g^−1^ (e.g., MOFs). **Table** **1** presents examples of some *SSA* values and pore volumes (*V*_p_) for different porous materials in these families.^[^ ^1^, ^2^, ^3^, ^4^, ^5^, ^6^, ^7^, ^8^, ^9^, ^10^ ^]^ To put this into perspective, some MOFs have more surface area of an American football field (5350 m^2^) per gram of material, e.g., DUT‐32 with 6411 m^2^ g^−1^.^[^ ^11^ ^]^

**Table 1:** Summary of specific surface areas (*SSA*) and pore volumes (*V*_p_) from the literature for a variety of aerogels, xerogels, chalcogels, metal–organic frameworks (MOFs), and zeolites. Materials are listed in descending order by material class in terms of *SSA* values

Material class	Material	*SSA* [m^2^ g^−1^]	*V*_p_ [cm^3^ g^−1^]	Ref.
Aerogel	Carbon aerogel	1040–1920	0.7–1.9	^[^ ^3^ ^]^
	Silica aerogel	820–1110	2.6–4.7	^[^ ^1^ ^]^
	Al‐Si‐O	940	2.4	^[^ ^2^ ^]^
	Na‐Al‐Si‐O	240–880	1.3–3.3	^[^ ^2^ ^]^
	Resorcinol‐formaldehyde	360–830	0.4–3.1	^[^ ^3^ ^]^
Xerogel	Carbon xerogel	600–850	1.4–1.6	^[^ ^5^ ^]^
	Silica xerogel	420–970	0.2–1.2	^[^ ^4^ ^]^
	Sn_2_S_3_	0.14	–	^[^ ^6^ ^]^
Chalcogel	Sn_2_S_3_ chalcogel	460	3.9	^[^ ^6^ ^]^
	PtGe_2_S_5_ chalcogel	420	3.1	^[^ ^6^ ^]^
MOF	DUT‐32	6411	2.91	^[^ ^11^ ^]^
	MOF‐5	2890	1.2	^[^ ^7^ ^]^
	ZIF‐8	1080	0.3	^[^ ^8^ ^]^
Zeolite	Mordenite	570	0.3	^[^ ^10^ ^]^
	ZSM‐5	410	0.1	^[^ ^10^ ^]^
	Zeolite A	120–240	0.2–0.5	^[^ ^9^ ^]^

The *SSA* parameter may be used to compare porous sorbents by normalizing the values on a mass basis, but it does not account for porous materials that are assembled using high‐density moieties containing heavy metals such as those used in fabricating chalcogen‐based aerogels (e.g., Ge_4_S_10_^4−^, SnS_4_^4−^, SnSe_4_^4−^), called chalcogels.^[^ ^12^ ^]^ Thus, in some cases, a correction factor can be used to renormalize the *SSA* values as a silica equivalent (*SSA*_eq_) value using Equation (1) where *SSA*_m_ is the measured specific surface area for the material of interest, $mw_{{\text{SiO}}_{2}}$ is the molecular weight of SiO_2_, and *mw*_x_ is the molecular weight of the material of interest normalized to two anions.^[^ ^13^, ^14^ ^]^ As an example, PtGe_2_S_5_ (*SSA*_*m*_ = 491 m^2^ g^−1^)^[^ ^15^ ^]^ would be normalized to Pt_0.4_Ge_0.8_S_2_ where *mw*_x_ = 200.3 g mol^−1^ and the corresponding *SSA*_eq_ is 1636 m^2^ g^−1^

(1) $$SSA_{\text{eq}}=\;\;SSA_{\text{m}}\;\text{*}\left(mw_{\text{x}}\text{/}mw_{{\text{SiO}}_{2}}\right)$$

**Figure 1:**
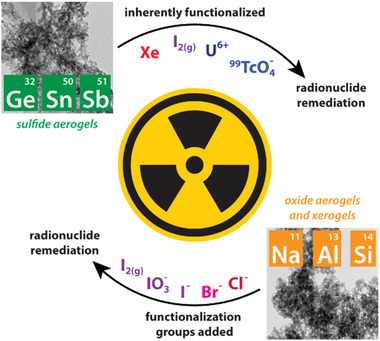
Overview of the material classes and some target contaminants discussed within the context of this progress report.

While previous reviews^[^ ^16^, ^17^, ^18^, ^19^, ^20^, ^21^ ^]^ have shown the applications where different porous materials can be used to capture and/or immobilize various contaminants, this paper focuses solely on functionalized aerogels and xerogels. Materials discussed herein include oxide‐based and sulfide‐based aerogels and xerogels as well as carbon‐based aerogel composites that have been demonstrated to capture a range of environmental contaminants including those in gaseous form [e.g., Hg_(g),_ I_2(g)_, Xe, Kr] as well as some in ionic form (e.g., Cd^2+^, Ce^4+^, Cs^+^, Cu^2+^, Fe^2+^, Hg^2+^, I^−^, IO_3_^−^, Kr, Pb^2+^, Rb^+^, Sr^2+^, ^99^Tc^7+^, U^6+^, Zn^2+^) (see **Figure** **1**). Descriptions of common synthesis methods utilized in the literature as well as some precursor selection guidelines will be provided for each class of sorbent.

## 2 Synthesis Methods

**Figure 2:**
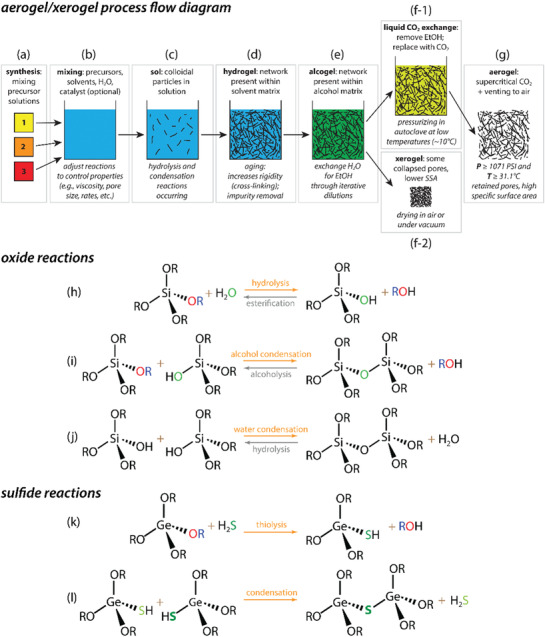
Process flow diagram for making g) aerogels or f‐2) xerogels starting with a) precursor solutions; additional stages include b) mixing precursors, c) the sol, d) hydrogels, e) alcogels, and f‐1) liquid CO_2_ exchange; Adapted with permission.^[^ ^2^ ^]^ Copyright 2017, American Chemical Society. h) Hydrolysis/esterification, i) alcohol condensation/alcoholysis, and j) water condensation/hydrolysis processes where “R” denotes a charged organic group, e.g., CH_3_^+^, C_2_H_5_^+^ (after Brinker^[^ ^22^ ^]^). Adapted with permission.^[^ ^2^ ^]^ Copyright 2017, American Chemical Society. k) Thiolysis and l) condensation of Ge(OR)_4_ metal alkoxide using H_2_S where “R” denotes a charged organic group, e.g., CH_3_^+^, C_2_H_5_^+^ (after Brock and Yu^[^ ^23^ ^]^).

Methods for producing aerogels and xerogels vary widely based on the target chemistry, but the processes involved are similar—some of these methods are briefly described in the process flow diagram shown in **Figure** **2**a–g. To start this process, the target chemistry must be selected. If the goal is to reach an oxide‐based chemistry, alkoxides are a typical selection of precursor, e.g., tetraethyl orthosilicate (TEOS) for building an Si—O network, described in Section 2.1. For building a chalcogen‐based gel network, an entirely different set of precursor chemicals must be used, described in Section 2.2. In both systems, a compatible solvent is used as a medium in which the reactions take place; in some cases, it is also used to dissolve precursor solids. However, for both oxide‐based and chalcogen‐based sol‐gel systems, precursors undergo a similar series of reactions (Figure 2h–l)^[^ ^22^, ^23^ ^]^ that result in interlinking chemical bonds between the precursors to create a *sol*, which is a series of colloidal particles in solution (Figure 2c). This mixture can be placed inside a container that will ultimately become the final shape of the gel in subsequent phases of the process. Once the sol is generated, a gel can form, but the gelation time can vary from minutes to months, depending on the chemistry of the precursors, solution pH, temperature, and the presence of a catalyst. The container selection is also important to make sure that the gel can be removed from the container at later stages without damaging the mechanical integrity of the product.

Once gelation occurs, if water is the medium, the product is called a *hydrogel* (Figure 2d). At this stage, it is common to allow the gel to remain in the medium for an extended time, called *aging*. Aging is a necessary step to allow for completion of hydrolysis and polycondensation reactions and to allow the gel to become more rigid. Once this process is complete, where the time varies for each gel chemistry, the reaction byproducts need to be removed through passive diffusion from the liquid medium within the gel matrix through a series of solvent exchanges. This typically involves water, such as 50/50 ethanol/water for oxide‐based chemistries or 50/50 formamide/water for chalcogel chemistries. The more times this process is conducted, the more effectively byproducts are removed, but additional rinses will increase the volume of waste produced. The larger the gel pieces are during this step, the more difficult this phase becomes.

Following this stage, the water must be removed though a series of exchanges with an organic solvent; typically, this solvent is ethanol. Again, this is accomplished through passive diffusion to dilute the water present within the gel matrix, and mixing is recommended during this step to help agitate the gels. If the solvent is completely exchanged to an alcohol, this product is called an *alcogel* (Figure 2e). At this stage, the sample can be dried (e.g., in air, under vacuum) to create a *xerogel* (Figure 2f‐2); this process can be destructive to the gel network and often results in significantly collapsed pore structures and reduced *SSA* values. Some things can be done to prevent pore structure collapse and that will be discussed in a later section. An alternative technique is to solvent exchange an alcogel to a polar organic solvent, such as hexane, prior to drying to reduce capillary forces.^[^ ^24^ ^]^

If the alcogel is then solvent exchanged with liquid CO_2_ in an autoclave to remove the alcohol (Figure 2f) using several rinses through passive diffusion, the CO_2_ can be taken to a supercritical state (i.e., ≥7.39 MPa *and* ≥31.1 °C) and vented slowly as a gas to produce an *aerogel* (Figure 2g). This process of allowing the CO_2_ to enter a supercritical state significantly reduces the surface energy and prevents the gel network from collapsing during this transition, allowing the gel to retain the *SSA* present within the alcogel phase. Examples of tools to aid in the processes of solvent exchange and critical point drying are shown in Figure S1 (Supporting Information).

### 2.1 Making Oxide‐Based Aerogels

#### 2.1.1 Precursor Selection and Target Reactions

**Table** **2** lists a series of different species that can be used to fabricate oxide gels and/or for usage in sol‐gel techniques that sometimes yield non‐gel products.^[^ ^2^, ^25^, ^26^, ^27^, ^28^, ^29^, ^30^, ^31^, ^32^, ^33^, ^34^, ^35^, ^36^, ^37^, ^38^, ^39^, ^40^, ^41^ ^]^ Some of these reactants are liquids at room temperature, some are solids, and some are dissolved (or suspended) in a secondary liquid, which is typically an alcohol (e.g., methanol, ethanol, isopropanol). The hydrolysis rates of metal alkoxides vary widely where some compounds are quite stable in air (e.g., TEOS) while others can hydrolyze readily in air [e.g., aluminum tri‐*sec*‐butoxide or Al(OBu^s^)_3_, sodium ethoxide or NaOEt] and require storage and handling in an inert atmosphere such as a nitrogen glovebox. Depending on the target chemistry, sometimes several options are available for a given bond addition such as tetraethyl orthosilicate (TEOS) or tetramethyl orthosilicate (TMOS) for adding Si—O moieties to an oxide‐gel network.

**Table 2:** Summary of various alkoxide precursor options and chemistries that they provide for the gel network, sorted alphabetically based on network cation charge (*M* *^n^* ^+^) from high (*n* = 5) to low (*n* = 1) oxidation states. Information provided here include the bond addition to the network, the additive to achieve this species, common abbreviations used for these additives in the literature, and references where these materials have been used to fabricate gel materials or as additives in sol‐gel processes

*M* *^n^* ^+^ (*n* = )	Network cation	Bond addition(s)	Additive (formula)	Common (or IUPAC) name	Common abbrev.	Ref.
+5	Nb^5+^	Nb—O	Nb(OC_2_H_5_)_5_	Niobium(V) ethoxide	NbOEt	^[^ ^25^ ^]^
	Ta^5+^	Ta—O	Ta(OC_2_H_5_)_5_	Tantalum(V) ethoxide	TaOEt	^[^ ^26^ ^]^
	V^5+^	O=V—O	VO(OC_3_H_7_)_3_	Vanadyl isopropoxide	VOPr	^[^ ^27^ ^]^
+4	Ge^4+^	Ge—O	Ge(OC_2_H_5_)_4_	Tetraethyl orthogermanate	GeOEt	^[^ ^28^ ^]^
	Si^4+^	Si—O	Si(OC_2_H_5_)_4_	Tetraethyl orthosilicate	TEOS	^[^ ^2^ ^]^
			Si(OCH_3_)_4_	Tetramethyl orthosilicate	TMOS	^[^ ^29^ ^]^
	Sn^4+^	Sn—O	Sn(OC_3_H_7_)_4_	Tin(IV) isopropoxide	Sn(IV)OPr	^[^ ^30^ ^]^
	Ti^4+^	Ti—O	Ti(OC_2_H_5_)_4_	Titanium(IV) ethoxide	TiOEt	^[^ ^31^ ^]^
	Zr^4+^	Zr—O	Zr(OC_3_H_7_)_4_	Zirconium(IV) propoxide	ZrOPr	^[^ ^32^ ^]^
+3	Al^3+^	Al—O	Al(OC_4_H_9_)_3_	Aluminum tri‐*sec*‐butoxide	Al(OBu^s^)_3_	^[^ ^2^ ^]^
	B^3+^	B—O	B(OC_2_H_5_)_3_	Triethyl borate	TEOB	^[^ ^33^ ^]^
			B(OCH_3_)_3_	Trimethyl borate	TMOB	^[^ ^34^ ^]^
	Sb^3+^	Sb—O	Sb(OC_2_H_5_)_3_	Antimony(III) ethoxide	SbOEt	^[^ ^35^ ^]^
	Y^3+^	Y—O	Y(OC_3_H_7_)_3_	Yttrium(III) tris(isopropoxide)	YOPr	^[^ ^36^ ^]^
+2	Ca^2+^	Ca—O	Ca(C_3_H_7_O_2_)_2_	Calcium 2‐methoxyethoxide	CaOMeEt	^[^ ^37^ ^]^
	Mg^2+^	Mg—O	Mg(OCH_3_)_2_	Magnesium methoxide	MgOMe	^[^ ^38^ ^]^
	Cu^2+^	Cu—O	Cu(OCH_3_)_2_	Copper(II) methoxide	CuOMe	^[^ ^39^ ^]^
+1	K^+^	K—O	KOC_2_H_5_	Potassium ethoxide	KOEt	^[^ ^40^ ^]^
			KOCH_3_	Potassium methoxide	KOMe	^[^ ^41^ ^]^
	Na^+^	Na—O	NaOC_2_H_5_	Sodium ethoxide	NaOEt	^[^ ^2^ ^]^
			NaOCH_3_	Sodium methoxide	NaOMe	^[^ ^2^ ^]^

The reactions that occur in the addition of water to an alkoxide are summarized in Figure 2h–j for a Si‐based alkoxide based on work by Brinker.^[^ ^22^ ^]^ These reactions include hydrolysis, alcohol condensation, water condensation, alcoholysis, and esterification. The differences between these reactions include the bonds that are formed and the byproducts that are released as a result.

Catalyst additions to these processes can be used to speed up the gelation time by accelerating the hydrolysis and polycondensation reactions. An array of catalysts have been reported for oxide gels from acids (e.g., HCl, H_2_SO_4_, HF, HNO_3_, and acetic acid or HOAc) to bases (e.g., KOH, NH_4_OH), as well as salts (e.g., KF).^[^ ^22^, ^42^ ^]^ A good example of the differences in gelation times for uncatalyzed reactions versus catalyzed reactions (i.e., 0.05:1 catalyst:TEOS, by mole) was provided by Pope and Mackenzie^[^ ^43^ ^]^ for comparing gelation times for TEOS hydrolyzed with 4 moles of water per mole of TEOS. They reported gelation times of 12 h for HF, 72 h for HOAc, 107 h for NH_4_OH, or >1000 h if uncatalyzed.^[^ ^43^ ^]^ It is important to fully remove the catalyst during solvent exchanging to prevent corrosion of autoclaves and other equipment at subsequent steps in the gel production process.

#### 2.1.2 Loading Getters onto Aerogel Scaffolds

**Figure 3:**
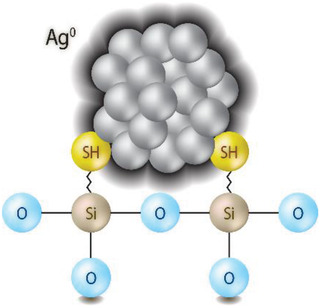
Cartoon showing Ag^0^ moieties attaching to thiol tethers using 3MPTS (see Section 2.1.2). Reproduced with permission.^[^ ^16^ ^]^ Copyright 2016, Elsevier.

Getters can be added to oxide aerogel scaffolds using a variety of techniques including ion exchange, impregnation, and functionalization. For instance, materials can be soaked in a solution containing a salt with the desired metal cation such as using AgNO_3_ for adding Ag^+^,^[^ ^44^ ^]^ soaked in a molten salt (e.g., AgNO_3_, 50/50 AgNO_3_/AgClO_3_ by mass),^[^ ^45^ ^]^ or by using (3‐mercaptopropyl) trimethoxysilane (3MPTS) to add thiol (—SH) tethers followed by soaking in AgNO_3_ (see **Figure** **3**).^[^ ^46^ ^]^ In the case of thiolation, the Ag links to the —SH tethers that are bound to an organic moiety connected to the silica backbone of the base aerogel material. The thiolation process is conducted by first hydrating the gels in a humid glass jar overnight, adding 3MPTS dropwise to the gels at ≈1.5 mL g^−1^ of unhydrated sample (the mass before hydration), loading the gels into a high‐pressure autoclave at 150 °C, loading the vessel with liquid CO_2_ at 24 MPa, and allowing this to sit for up to 2 d in supercritical CO_2_. Following the soak at 150 °C, the CO_2_ can be vented as a gas and the gel material removed for the subsequent step of adding the getter (e.g., Ag^+^ with AgNO_3_).

Ion exchange processes are commonly practiced for replacing cations within zeolite frameworks and this same approach can be applied to aerogel/xerogel chemistries. The selected getter loading process will depend on the gel chemistry, chemical compatibility with the available options, and the getter chemistry of choice. For instance, each getter will have different solubilities in different solvents, where some solvents could destroy (e.g., dissolve) the scaffold network; dissolving the getter additive in the base solvent can drastically change the pH of the solution.^[^ ^47^ ^]^ For example, acidic solutions can dissolve silica networks.

### 2.2 Making Chalcogen‐Based Aerogels

Chalcogenide aerogels, also called chalcogels, can be prepared through different, albeit similar, methods to oxide‐based gels but generally with non‐oxide precursors. These processes include 1) thiolysis of metal alkoxides with H_2_S, 2) aggregation of nanoparticles, or 3) metathesis reactions (i.e., chemical linkage of chalcogenide clusters with interlinking metal ions). Several different example chemistries for each synthesis option are provided in **Table** **3**,^[^ ^12^, ^13^, ^14^, ^15^, ^48^, ^49^, ^50^, ^51^, ^52^, ^53^, ^54^, ^55^, ^56^, ^57^, ^58^, ^59^, ^60^, ^61^, ^62^ ^]^ and additional information can be found in a previous review by Brock and Yu^[^ ^23^ ^]^ as well as papers by Krebs^[^ ^63^ ^]^ and Kanatzidis et al.^[^ ^12^, ^55^, ^59^, ^62^ ^]^ The chemistries available for study are broad with a wide range of applications discussed in later sections. Chalcogel compositions include chemistries of S‐based,^[^ ^13^ ^]^ Se‐based,^[^ ^64^ ^]^ and Te‐based^[^ ^65^ ^]^ networks.

**Table 3:** Summary of different types of commonly studied chalcogel chemistries. Note that solvents used are not included herein and the reader is referred to the primary literature for the full synthesis procedures

Method	Chemistry (family)	Primary precursor(s)^a)^	Interlinking metal(s), M	Ref(s).
Alkoxide thiolation	Ge‐S	Ge(OC_2_H_5_)_4_, H_2_S	Ge^4+^	^[^ ^48^ ^]^
	La‐S	La(NSi_2_C_6_H_18_)_3_, H_2_S	La^3+^	^[^ ^49^ ^]^
	Nb‐S	Nb(OC_2_H_5_)_5_, H_2_S	Nb^5+^	^[^ ^50^ ^]^
	Ti‐S	Ti(OC_3_H_7_)_4_, H_2_S	Ti^4+^	^[^ ^50^ ^]^
	W‐S	W(OC_2_H_5_)_6_, H_2_S	W^6+^	^[^ ^51^ ^]^
	Zn‐S	Zn(OC_4_H_9_)_2_, H_2_S	Zn^2+^	^[^ ^52^ ^]^
Nanoparticle aggregation	CdS	Cd(NO_3_)_2_, Na_2_S	Cd^2+^	^[^ ^53^ ^]^
	CdSe	Cd(NO_3_)_2_, Na_2_Se	Cd^2+^	^[^ ^53^ ^]^
	PbS	Pb(NO_3_)_2_, Na_2_S	Pb^2+^	^[^ ^53^ ^]^
	ZnS	Zn(OAc)_2_, Na_2_S	Zn^2+^	^[^ ^53^ ^]^
Metathesis reactions	Pt(Ge,Sn)(S,Se)	Ge_4_(S,Se)_10_^4−^, Sn_4_Se_10_^4−^, Sn_2_Se_6_^4−^, Sn(S,Se)_4_^4−^	Pt^2+^	^[^ ^13^, ^14^, ^15^ ^]^
	(Mo,W)‐M‐S	(Mo,W)S_4_^2−^	Co^2+^, Ni^2+^	^[^ ^54^, ^55^ ^]^
	(K,Na)(Sb,As)‐Fe‐(S,Te)	AsS_3_^3−^, SbS_3_^3−^, SbTe_3_^3−^	Fe^2+^, K^+^, Na^+^	^[^ ^56^, ^57^ ^]^
	(Sn,Sb)‐M‐(S,Se)	Sn_2_(S,Se)_6_^4−^, Sn(S,Se)_4_^4−^, SbSe_4_^3−^	Sn^2+^, Sb^3+^	^[^ ^12^, ^58^ ^]^
	Zn‐Sn‐S	SnS_4_^4−^, Sn_2_S_6_^4−^, Sn_4_S_10_^4−^	Zn^2+^	^[^ ^59^ ^]^
	Fe‐Sn‐S	Fe_4_S_4_ *^m^* ^−^, Sn_2_S_6_^4−^	Fe_4_S_4_ *^m^* ^−^, Sn_2_S_6_^4−^	^[^ ^60^ ^]^
	Fe‐M‐Sn‐S	Fe_4_S_4_ *^m^* ^−^, Sn_2_S_6_^4−^	Zn^2+^, Sn^2+^, Ni^2+^, Co^2+^	^[^ ^61^ ^]^
	Mo‐Co‐M‐S	MoS_4_^2−^	Co^2+^, Pb^2+^, Cd^2+^, Pd^2+^, Cr^3+^, Bi^3+^	^[^ ^62^ ^]^

a) OAc denotes acetate (or CH_3_COO^−^).

#### 2.2.1 Alkoxide Thiolysis

The thiolysis synthesis route for making chalcogels is similar to that described in Section 2.1 for producing oxide‐based aerogels using metal alkoxides. In this case, the same metal alkoxides can be used but, instead of adding H_2_O to hydrolyze the alkoxides and build an oxide‐based network through —O— bridges, H_2_S_(g)_ is bubbled through the solution; the effect of adding H_2_S is similar to that of adding H_2_O, but adding H_2_S results in —S— bridges. This process is shown schematically in Figure 2k,l, which is based off a description provided by Brock and Yu.^[^ ^23^ ^]^

This same process can also be used to create —Se— or —Te— bridges by exposing materials to H_2_Se_(g)_ or H_2_Te_(g)_, respectively, but these gases are extremely toxic. Alternative methods have been employed by adding metal salts and Se^0^ or Te^0^ to a material followed by heating to precipitate crystals with a matrix such as CdSe or CdTe.^[^ ^66^ ^]^

#### 2.2.2 Nanoparticle Aggregation

For nanoparticle aggregation, metal chalcogenides have been shown to interconnect if prepared through room‐temperature reverse‐micellar strategies or high‐temperature arrested‐precipitation. The chemistries demonstrated with this technology include CdS, CdSe, PbS, and ZnS. It was observed in the work by Mohanan and Arachchige^[^ ^53^ ^]^ that these materials tended to have short‐range order evidenced by semicrystalline nanoparticles observed with transmission electron microscopy (TEM). The *SSA* values for gels produced by Mohanan and Arachchige^[^ ^53^ ^]^ with this technique ranged from 120–250 m^2^ g^−1^.

In addition to this work, chalcogels produced through nanoparticle agglomeration can be ion exchanged to produce even new chemistries. In a study by Yao et al.,^[^ ^64^ ^]^ CdSe gels were exchanged with Ag^+^ using AgNO_3_ dissolved in methanol to yield Ag_2_Se aerogels. The starting CdSe gel had an *SSA* of 133 m^2^ g^−1^ and, after exchange, the Ag_2_Se aerogel had an *SSA* of 111 m^2^ g^−1^.

#### 2.2.3 Metathesis Reactions: Chemical Linkage of Clusters and Interlinking Metals

**Figure 4:**
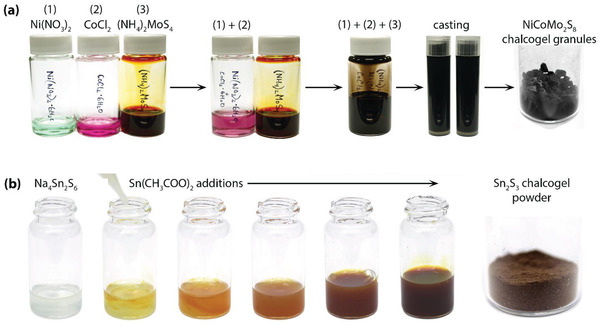
Precursor mixing and casting for making a) CoNiMo_2_S_8_ and b) Sn_2_S_3_ chalcogels in formamide followed by gel production after critical point drying (preceded by solvent exchanges, not shown). The graphics for (b) were reproduced with permission.^[^ ^68^ ^]^ Copyright 2014, American Chemical Society.

Producing chalcogels using the chalcogenide clusters and interlinking metal ions is a fairly recent discovery^[^ ^13^, ^14^ ^]^ that opens up more options and chemical flexibility than previous methods for making new chalcogel chemistries ranging from simple to complex. The available list of clusters is still growing as new chemistries can be synthesized following existing procedures. Some of the more well‐studied chemistries include Ge‐(S,Se), Sn‐(S,Se), Mo‐S, and W‐S systems. In most cases, the chalcogenide clusters are not commercially available and must be synthesized. For instance, one of the salts used to provide Ge_4_S_10_^4−^, [(CH_3_)_4_N]_4_Ge_4_S_10_, is made hydrothermally from (CH_3_)_4_NOH, Ge powder, and S powder where the product solution can be filtered and a solvent added (e.g., acetone) to induce precipitation/crystallization toward the target compound.^[^ ^67^ ^]^ Common solvents for dissolving these precursors include water and formamide. Two examples, which are shown in **Figure** **4** are the synthesis of CoNiMo_2_S_4_ and Sn_2_S_3_ chalcogels described elsewhere.^[^ ^6^, ^55^, ^68^, ^69^ ^]^ For CoNiMo_2_S_4_, separate solutions of Ni(NO_3_)_2_, CoCl_2_, and (NH_4_)_2_MoS_4_ are dissolved in formamide, combined and mixed, and then casted into polypropylene vials to undergo gelation. For Sn_2_S_3_ chalcogel synthesis, Na_4_Sn_2_S_6_ ·14H_2_O and Sn(II) acetate are separately dissolved in formamide, combined and mixed, and then cast to undergo gelation.

## 3 Iodine Capture with Metal‐Functionalized Aerogels and Xerogels

### 3.1 Silver‐Functionalized Silica Aerogel

Silver‐functionalized silica aerogels (Ag^0^SA) are a relatively new class of iodine sorbents that are being evaluated by Matyáš et al.^[^ ^46^, ^70^ ^]^ to replace silver mordenite (Ag^0^Z) in nuclear fuel reprocessing off‐gas schemes proposed by the U.S. Department of Energy Office of Nuclear Energy (DOE‐NE); note that DOE‐NE currently considers Ag^0^Z as the baseline technology for I_2(g)_ capture. The Ag^0^SA sorbent has documented decontamination factors (DFs) upward of 10000 in laboratory tests of dissolver off‐gas streams, where DF is a metric for determining the effectiveness of the sorbent to remove a contaminant of interest from a stream (sometimes containing other species that might compete with the binding sites).^[^ ^46^, ^71^ ^]^ The DF parameter is defined in Equation (2) where CE is the capture efficiency (e.g., if 99.99% is captured, CE = 0.9999 and DF = 10000).^[^ ^16^ ^]^ The process for synthesizing these materials consists of several steps: 1) commercially available silica aerogel granules are heat‐treated to remove hydrophobic silyl functional groups from the aerogel surfaces, 2) a thiol (i.e., —SH) layer is added to the gel using the process mentioned above with 3MPTS (*thiolation*; see Section 2.1.2), 3) silver ions (i.e., Ag^+^) are added to the gel through chemisorption to the sulfur in the thiol groups (i.e., *Ag‐impregnation*; —S—Ag), and 4) the silver is reduced under a flowing stream of H_2_/Ar gas (i.e., *Ag‐functionalization*; —SH + Ag^0^; see Figure 3. Upon I_2(g)_ capture, AgI crystals are formed within the composite sorbent.

(2) $$\text{DF}\;\;=\;\;1\text{/}\left(1\;\;-\;\;\text{CE}\right)$$

Some of the benefits of the Ag^0^SA over the Ag^0^Z include a higher capacity for iodine (i.e., ≈480 mg g^−1^, ≈3–4× greater than Ag^0^Z)^[^ ^46^, ^70^, ^71^ ^]^ and a higher functionality after aging in NO*_x_* atmosphere and high‐humidity environments (both of which could be observed in a nuclear fuel reprocessing facility). Additionally, Ag^0^SA has thiol groups that help prevent oxidation of Ag^0^, a process that renders the sorbent less effective, and it can be consolidated into a multiphase waste form containing AgI particles encapsulated within a glassy silica matrix.

In a recent paper by Matyáš et al.,^[^ ^70^ ^]^ X‐ray photoelectron spectroscopy analysis on base sorbents and sorbents loaded with I_2(g)_ revealed some key insights into the way that this sorbent works. For instance, when the sorbents were exposed to a 1 vol% NO_(g)_ atmosphere during an aging experiment, the Ag^0^ nanoparticle sizes increased and free sulfate was observed on the surfaces of the material due to oxidized thiol groups (i.e., S^2+^ → S^6+^). These changes to the material prevented full utilization of the Ag^0^ for I_2(g)_ capture. In some cases, Ag_2_SO_4_ was observed, providing evidence that the thiol layers help act as a buffer to prevent oxidation of the Ag^0^, because the Ag_2_SO_4_ crystals tend to surround the Ag^0^ nanocrystals and act as a barrier to further Ag^0^ oxidation. Unfortunately, this barrier layer lowers the Ag^0^ accessibility during iodine capture experiments, resulting in decreased I_2(g)_ sorption capacity. Studies demonstrated that the sorbent could be regenerated under a reducing gas stream to revert the oxidized Ag^+^ back to Ag^0^ and oxidized SO_4_^2−^ back to S^2+^. After aging, the Ag^0^SA showed a reduced I_2(g)_ sorption capacity of 43 relative%, but this was in comparison to a 85 relative% reduction for the Ag^0^Z,^[^ ^72^, ^73^ ^]^ further accentuating the benefits of the Ag^0^SA sorbent.

Since the initial discovery of these materials in 2010, several improvements have been made over the last decade. One of the most important improvements has been the increased mechanical rigidity, or the reduction in friability, of the base sorbent using heat treatments and additives during the sorbent preparation process (e.g., TEOS). It was discovered that heat treating the silica aerogel granules at 700–800 °C yielded granules with high resistance to damage and the granules retained high *SSA* (≈950 m^2^ g^−1^). Another important development is the consolidation of the material into a dense waste form. After iodine loading, these materials can be readily consolidated using a variety of techniques with negligible iodine loss including hot uniaxial pressing, spark plasma sintering, and hot isostatic pressing.^[^ ^46^, ^74^, ^75^ ^]^

Finally, in a different study,^[^ ^76^ ^]^ the Ag^0^SA sorbents were demonstrated to remove iodate (i.e., IO_3_^−^) and iodide (i.e., I^−^) from aqueous solutions; iodine can be present as these species in radionuclide‐contaminated groundwater as well as in caustic scrubbing solutions from radiological off‐gases. The efficacy of this sorbent to remove I^−^ was demonstrated in a variety of different solutions including some with competing halide ions Br^−^ and Cl^−^ as well as the removal of IO_3_^−^ after a reduction to I^−^ was observed. In the presence of Cl^−^, Br^−^, and I^−^, the Ag^0^SA showed preferential removal in the order of I^−^ > Br^−^ > Cl^−^. For I^−^ removal, a distribution coefficient (*K*_d_) of 1.46 × 10^4^ in off‐gas simulant was demonstrated as well as a *K*_d_ of 1.5 × 10^4^ in pure deionized water (DIW); *K*_d_ is defined in Equation (3) where *c*_i_ is the initial concentration of species in solution, *c*_t_ is the concentration after a time interval, *V* is the volume of solution (mL), and *m* is the mass of solid. Also, the kinetics of I^−^ removal were faster than that of IO_3_^−^ removal; 98% of I^−^ was removed within 1 h of contact in DIW (*c*_i_ = 0.044 mmol L^−1^) whereas 94% of IO_3_^−^ was removed after contact for ≈12 d in DIW (first‐order sorption rate = 9.35 × 10^−3^ h^−1^)

(3) $$K_{\text{d}}=\;\;\left(\frac{c_{\text{i}}-c_{\text{t}}}{c_{\text{t}}}\right)\;\;\left(\frac{V}{m}\right)$$

### 3.2 Aluminosilicate Aerogel and Xerogels for Iodine Capture

#### 3.2.1 Silver‐Loaded Aluminosilicates for Iodine Capture

Silver‐loaded aluminosilicate aerogels were developed as follow‐on work to the silver‐functionalized silica aerogels.^[^ ^2^ ^]^ For this work, Na‐Al‐Si‐O and (Na‐free) Al‐Si‐O aerogels were fabricated from metal alkoxides including TEOS, Al(OBu^s^)_3_, NaOMe, and NaOEt (see Table 2 for definitions) with the target compositions of NaAlSiO_4_ and AlSiO_3.5_, respectively. For some formulations, an HOAc catalyst was implemented to reduce gelation times. A series of different parameters were evaluated during the synthesis including the Na‐precursor, cosolvent alcohols (e.g., methanol, ethanol, isopropanol) utilized to suspend the Na and Al precursors, batch size, mixing times, and the mixing environment (i.e., in air or within a nitrogen glovebox). Following gelation, gels were removed from the casting vials, diced, and rinsed with 50/50 ethanol/DIW (10×), followed by solvent exchanges to pure ethanol (10×), exchanged in liquid CO_2_ (10×), then the vessel was taken to the supercritical CO_2_ state, and the CO_2_ was vented as a gas, yielding aerogels. Several batches resulted in high *SSA* values ranging from 517 to 882 m^2^ g^−1^ for the Na‐Al‐Si‐O aerogels and up to 942 m^2^ g^−1^ for Al‐Si‐O aerogels. Examples of each alcogel and aerogel are shown in Figure S2 (Supporting Information).

**Figure 5:**
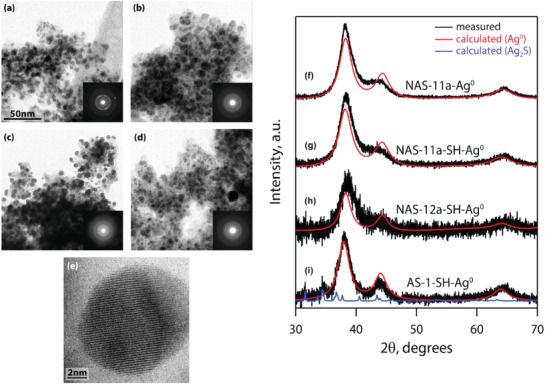
a–e) TEM images and f–i) powder X‐ray diffraction patterns from Ag‐functionalized Na‐Al‐Si‐O aerogels and an Al‐Si‐O aerogel (AS‐1‐SH‐Ag^0^). These data show that the Ag present within these gels is crystalline Ag^0^. Adapted with permission.^[^ ^2^ ^]^ Copyright 2017, American Chemical Society.

Following gel synthesis, different approaches were utilized to load Ag into the gel network including: 1) soaking as‐made (AM) gels in AgNO_3_ solutions followed by drying (*Ag‐impregnation*), 2) reducing the Ag^+^ in gels that underwent Ag‐impregnation to Ag^0^ under a flowing stream of 2.7%H_2_/Ar gas (*Ag‐reduction*), and 3) thiolating gels that experienced Ag‐impregnation and Ag‐reduction (*Ag‐functionalization*). The appearances of these materials afterward were notably different in color (note that pictures of the materials can be found in the original work).^[^ ^2^ ^]^ During chemical analysis of the gels after Ag‐impregnation, the Na concentrations in the Na‐Al‐Si‐O base gels were really low, and nearly 0 mass% in some cases. After additional Ag‐exchange testing, the Ag^+^ was found to replace the Na^+^ in the gel network. After analyzing the Al‐Si‐O gels (those without Na), it was clear that the Ag‐content was much lower at 12 at% versus 35 at% for Ag‐functionalized gels (starting from Na‐Al‐Si‐O gels containing Na). Analyses with TEM and powder X‐ray diffraction (PXRD) on functionalized gels revealed discrete and consistently sized Ag^0^ nanoparticles present within the gels, which ranged between ≈3 and 14 nm in diameter (see **Figure** **5**). Iodine loadings were conducted in a polymer autoclave containing the sample and 99.99% pure iodine crystals at 150 °C over the course of 24 h. To remove physisorbed iodine, the iodine was removed from the container, and the samples were placed back into the oven without the iodine for ≈1 h.

**Figure 6:**
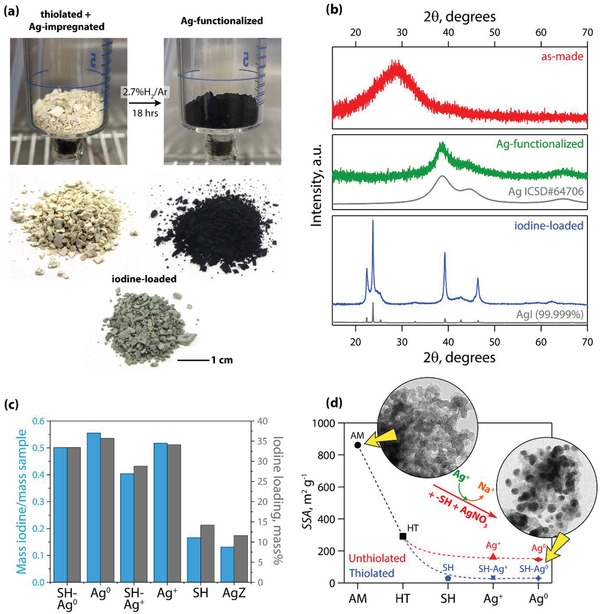
a) Pictures of thiolated/Ag‐impregnated and Ag‐functionalized Na‐Al‐Si‐O aerogels within the Ag‐reduction chamber (under flowing H_2_/Ar); pictures of thiolated/Ag‐impregnated, Ag‐functionalized, and iodine‐loaded Na‐Al‐Si‐O aerogels. b) PXRD patterns for as‐made, Ag‐functionalized, and iodine‐loaded Na‐Al‐Si‐O aerogels; a refinement of Ag (ICSD No. 53761)^[^ ^77^ ^]^ is shown in addition to the PXRD pattern of 99.999% pure AgI. c) Iodine uptake of various samples including thiolated/Ag‐functionalized (SH‐Ag^0^), Ag‐reduced (Ag^0^), thiolated/Ag^+^‐impregnated (SH‐Ag^+^), thiolated (SH), and silver mordenite as a comparison (shown as AgZ). d) Comparison of *SSA* values for samples at different stages during the synthesis process including as‐made (AM), heat‐treated at 350 °C for 30 min (HT), SH, Ag^+^, Ag^0^, SH‐Ag^+^, and thiolated/Ag‐reduced (SH‐Ag^0^ or Ag‐functionalized); included are TEM micrographs showing the as‐made Na‐Al‐Si‐O and Ag‐exchanged (Ag‐Al‐Si‐O) aerogels. Adapted with permission.^[^ ^2^ ^]^ Copyright 2017, American Chemical Society.

**Figure** **6**a provides visual appearances for the thiolated and Ag^0^‐functionalized Na‐Al‐Si‐O aerogels both inside and outside the H_2_/Ar Ag‐reduction apparatus. The PXRD patterns of the as‐made, Ag‐functionalized, and iodine‐loaded Na‐Al‐Si‐O aerogels are provided in Figure 6b. The AM aerogel had a broad diffraction peak centered around 29° 2θ, which is indicative of an amorphous network without long‐range order. The Ag‐functionalized aerogel did not have the same diffraction peak as the as‐made aerogel, but rather had some broad peaks centered around 38°–46° 2θ and 65° 2θ, which corresponded well with crystalline Ag^0^ (*Fm* $\bar{3}$ *m*, ICSD No. 53761)^[^ ^77^ ^]^ with an average crystallite size of 3.2 nm. The PXRD pattern for the iodine‐loaded aerogel showed very strong diffraction peaks for AgI as verified by a match with pure AgI and contained a mixture of hexagonal (*P*6_3_ *mc*, ICSD No. 62790)^[^ ^78^ ^]^ and cubic (*F* $\bar{4}$3*m*, ICSD No. 61542)^[^ ^79^ ^]^ crystalline morphologies.

The iodine‐loading capacities for these materials were rather high based on mass uptake and confirmed with scanning electron microscopy (SEM) and energy‐dispersive X‐ray spectroscopy (EDS) analysis showing high Ag‐utilization (I:Ag molar ratio). This means that the iodine loadings were consistent with the Ag‐loadings. The loadings based on mass uptake on a $m_{\text{I}}\;m_{\text{s}}^{-1}$ basis (mass of iodine per mass of starting sample) are shown along with the mass% of iodine measured in the sample via SEM‐EDS analysis for comparison in Figure 6c. Also included for comparison are data collected for Ag^0^Z. The iodine loadings ($m_{\text{I}}\;m_{\text{s}}^{-1}$) for Ag‐impregnated (Ag^+^), Ag‐reduced (Ag^0^), thiolated/Ag‐impregnated (—S—Ag^+^), and thiolated/Ag‐reduced (—SH + Ag^0^ or Ag‐functionalized) were 0.517, 0.555, 0.404, and 0.501 $m_{\text{I}}\;m_{\text{s}}^{-1}$, respectively. The iodine loadings were lower for thiolated versus unthiolated gels in each dataset (e.g., thiolated Ag‐impregnated vs Ag‐impregnated) and this is partly based on the added organic moieties present from the 3MPTS functionalization step (i.e., a diluent). It is also clear that the process of reducing the Ag from Ag^+^ to Ag^0^ has an added effect of increased iodine loadings from 7% to 24% for unthiolated and thiolated aerogels, respectively.

Based on these iodine loading data, extra processing steps could be avoided to yield a high‐functioning sorbent without utilizing thiolation and Ag‐reduction (Ag^+^ → Ag^0^). This expedites the sample preparation time and produces a highly functional base sorbent. However, there is also an added benefit to avoiding these steps as they decrease *SSA* values (Figure 6d). The most detrimental process step for lowering the *SSA* values is the heat‐treatment process (350 °C for 30 min in air) at the front end that is used to strengthen the gel network so that it does not collapse during the thiolation and/or Ag^+^‐impregnation steps. This heat treatment was also adopted for unthiolated gel processing. This step could be avoided but would likely result in gel damage during subsequent steps such as the Ag‐impregnation step. The second most detrimental step resulting in lowered *SSA* values was the thiolation process.

Following the 2017 study,^[^ ^2^ ^]^ additional comparisons were made in a 2020 study^[^ ^80^ ^]^ between Ag‐loaded aluminosilicate aerogels, heat‐treated aerogels, and xerogels. A critical difference between the 2020 study^[^ ^80^ ^]^ and the 2017 study^[^ ^2^ ^]^ is that the new work utilized a simplified process. For instance, no Ag reduction (using H_2_/Ar) and no thiolation were implemented, and xerogels were introduced into the process. Based on the 2017 study,^[^ ^2^ ^]^ iodine loadings were still determined to be high when the Ag^+^ was exchanged into the base material (0.517 $m_{\text{I}}\;m_{s}^{-1}$) without reducing the silver to Ag^0^.

Here, similar base materials in the alcogel state (see Figure 2e) were either converted to aerogels in a critical point dryer (Figure 2g) or dried in a vacuum desiccator to yield xerogels (Figure 2f‐2). Another difference in this study was that the xerogels retained nearly the full *SSA* (i.e., 531 m^2^ g^−1^) as the aerogel counterparts (598 m^2^ g^−1^) due to some process improvements based on the 2017 study.^[^ ^2^ ^]^ These improvements included a longer aging time for the alcogels (several months vs a few days) and ethanol removal under vacuum within a desiccator instead of drying them in air, where drying in air is a more energetic process. After Ag^+^‐exchange, the xerogels retained higher *SSA* values (i.e., 240 m^2^ g^−1^) compared to the aerogels that were not heat treated (i.e., 120 m^2^ g^−1^) and even the heat‐treated aerogels (i.e., 150 m^2^ g^−1^). Note that the heat treatment process was performed only on some of the aerogels to improve the mechanical rigidity of the base material (as discussed previously).

The more important comparison is in the iodine uptake values for these materials. The aerogels, heat‐treated aerogels, and xerogels showed iodine uptake values of 0.405, 0.377, and 0.327 $m_{\text{I}}\;m_{\text{s}}^{-1}$, respectively. These values are not as high as those achieved in the 2017 study,^[^ ^2^ ^]^ but the base materials differed in 2020 study;^[^ ^80^ ^]^ this difference was attributed to batch‐to‐batch variability and differences in aging times used between the different sets of experiments.

The main goal of the 2020 study^[^ ^80^ ^]^ was to evaluate whether or not the iodine uptake of Ag‐loaded aerogels and xerogels differed significantly. Eliminating extra processing steps during gel synthesis drastically simplified the process and allowed for the scale‐up of sorbent production volumes. Since aerogel production requires an autoclave, this adds additional pressure hazards to the process, but also makes scale‐up more difficult and expensive than it is for xerogel production. Finally, the xerogels are quite mechanically robust, especially compared to the base aerogels and even the heat‐treated aerogels.

#### 3.2.2 Ag‐Free Aluminosilicate Aerogels and Xerogels for Iodine Capture

A follow‐up study^[^ ^47^ ^]^ to the Ag‐loaded aluminosilicate study was conducted to evaluate non‐Ag getters in aluminosilicate aerogels and xerogels. The main driver for this study was to find alternative getters to Ag because Ag is a hazardous material controlled in the United States by the Environmental Protection Agency under the Resource Conservation and Recovery Act,^[^ ^81^ ^]^ complicating the waste form disposal process. Also, Ag is also a precious metal, making large‐scale sorbent production an expensive venture.

In this study, several different getters materials were evaluated based on thermodynamic predictions from metal‐iodide complexation ($\Delta G_{\text{f},\text{I}}^{{}^{\circ}}$) using Gibb's free energies compared to the metal‐oxide complexation ($\Delta G_{\text{f},\text{O}}^{{}^{\circ}}$) with HSC Chemistry (v9, Outotec, Finland). Calculations performed as part of that study showed that only 14 species in the database showed a more favorable metal‐iodide complexation over the metal‐oxide complexation including ($\Delta G_{\text{f},\text{I}}^{{}^{\circ}}$ < $\Delta G_{\text{f},\text{O}}^{{}^{\circ}}$) including: Cs^+^ (*T*_all_), K^+^ (*T*_all_), Rb^+^ (*T*_all_), Ag^+^ (*T*_all_), Ba^2+^ (*T*_all_), Sr^2+^ (25 °C ≤ *T* ≤ 325 °C), Hg^+^ (*T*_all_), Hg^2+^ (*T*_all_), Tl^+^ (*T* ≥ 400 °C), Eu^2+^ (25 °C ≤ *T* ≤ 400 °C), Pb^2+^ (25 °C ≤ *T* ≤ 100 °C), Pd^2+^ (*T*_all_), Pt^2+^ (25 °C ≤ *T* ≤ 150 °C), and Pt^4+^ (*T* = 25 °C).

**Figure 7:**
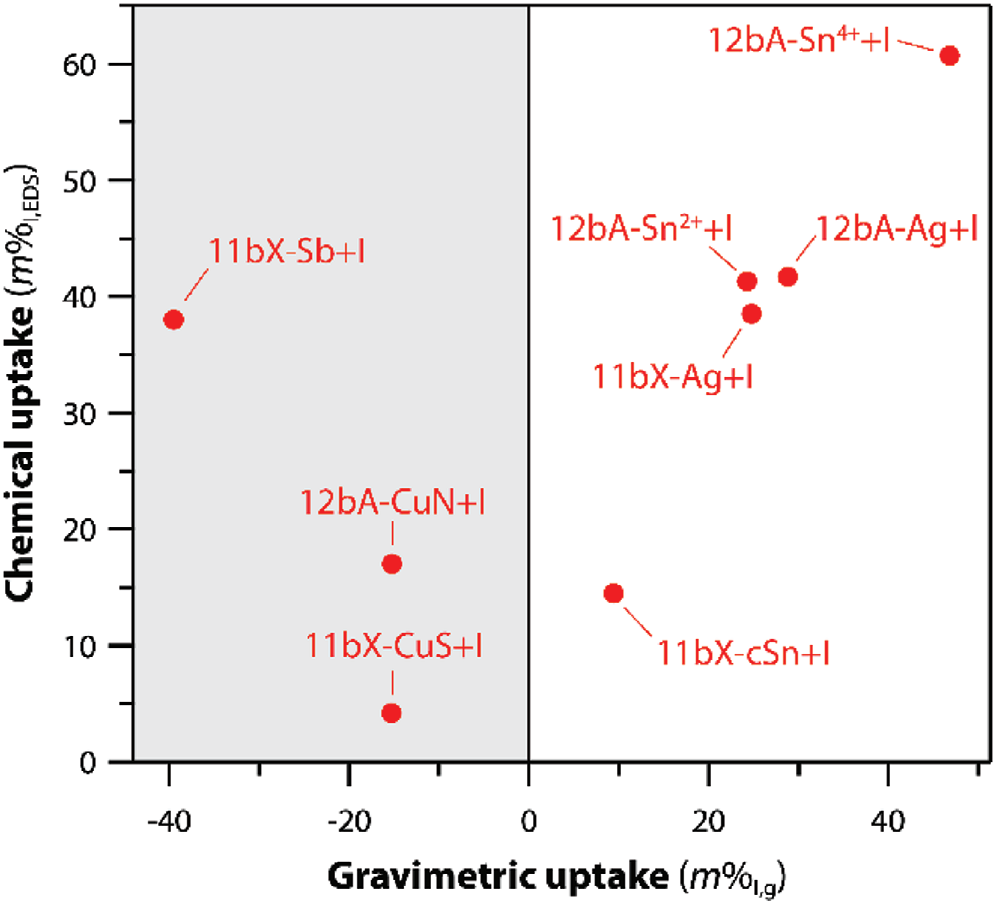
Comparison of chemical uptake based on EDS data (*m*%_I,EDS_) and gravimetric uptake (*m*%_I,g_). The left side of the figure (shaded in gray) shows data where negative gravimetric uptake and positive chemical uptake were observed. Reproduced with permission.^[^ ^47^ ^]^ Copyright 2020, American Chemical Society.

Some of these species were incorporated into the sodium aluminosilicate scaffold using ion exchange processes with different solvent systems and the final selection of getters included Cs^+^, Cu^2+^, Fe^3+^, K^+^, Li^+^, Rb^+^, Sb^3+^, Sn^2+^, and Sn^4+^ (in addition to Ag^+^), based on promising data in the literature.^[^ ^2^, ^6^, ^68^, ^69^, ^82^, ^83^, ^84^, ^85^ ^]^ The ion exchange process was typically depicted by the exchanged gel showing a loss of Na^+^ to the solution during the exchange as the target cations were loaded into the scaffold. The most effective materials at capturing iodine based on gravimetric uptake (measured by mass uptake) and chemical uptake (quantified by EDS analysis) were Cu^2+^ (CuSO_4_), Sn^2+^ [Sn(II) acetate], and Sn^4+^ [i.e., colloidal SnO_2_, Sn(IV) acetate] [in addition to Ag^+^ (AgNO_3_)] (see **Figure** **7**); note that the species in parenthesis are the salts added during ion exchange. More work is needed to fully understand the mechanisms of these exchanges for the different getter systems.

## 4 Chalcogen‐Based Aerogels (Chalcogels) for Radionuclide Capture

The two primary theories to explain the high affinity of chalcogels for capturing I_2(g)_ and heavy metals are centered around physisorption and chemisorption. From a physisorption perspective, the selective affinity of the chalcogen‐based aerogel backbone for I_2(g)_ can be explained by Pearson's hard/soft acid‐base (HSAB) principle.^[^ ^86^, ^87^ ^]^ The HSAB principle evaluates electron‐pair donating Lewis bases and electron‐pair accepting Lewis acids to predict complexation behaviors of cations and ligands.^[^ ^86^ ^]^ To compare species, the chemical hardness (η) can be calculated using the ionization energy (*I*) and electron affinity (*A*) for a given species “s” using Equation (4). If two species have similar chemical hardness values, where one is a Lewis acid and the other is a Lewis base, the HSAB principle predicts that these two species will share an affinity. **Table** **4** presents some different values for a range of Lewis acids and bases as well as some neutral species.^[^ ^87^, ^88^, ^89^, ^90^ ^]^ These data suggest that the chalcogen affinity for I_2(g)_ would rank in the order of Te > Se > S, but to date, only S‐based chalcogels have been evaluated for this application

(4) $$\eta_{\text{s}}=1\text{/}2\left(I_{\text{s}}-\;\;A_{\text{s}}\right)$$

**Table 4:** Tabulated values of *I*_s_, *A*_s_, χ_s_, and η_s_ for various species, “s,” sorted by η_s_ from hard to soft. Here, χ denotes the absolute electronegativity

Acid/base	Species	*I*_s_	*A*_s_	χ_s_	η_s_	Ref(s).
Acid	K^+^	31.63	4.34	17.99	13.65	^[^ ^87^, ^88^ ^]^
	Zn^2+^	39.7	17.96	28.8	10.8	^[^ ^87^ ^]^
	HCl	12.7	−3.3	4.7	8.0	^[^ ^89^ ^]^
	Hg^2+^	34.2	18.75	26.5	7.7	^[^ ^87^ ^]^
	U^4+^	45.77	31.06	38.415	7.4	^[^ ^90^ ^]^
	CO_2(g)_	13.8	0	6.9	6.9	^[^ ^87^ ^]^
	Cu^+^	20.3	7.72	14.0	6.3	^[^ ^87^ ^]^
	HI(g)	10.5	0.0	5.3	5.3	^[^ ^89^ ^]^
	HNO_3_	11.03	0.57	5.8	5.2	^[^ ^89^ ^]^
	I_2(g)_	9.3	2.6	6.0	3.4	^[^ ^87^ ^]^
Neutral	CH_3_I	9.5	0.2	4.9	4.7	^[^ ^89^ ^]^
	Cl_2(g)_	11.6	2.4	7.0	4.6	^[^ ^89^ ^]^
Base	S	10.36	2.08	6.22	4.12	^[^ ^87^ ^]^
	Se	9.75	2.02	5.89	3.86	^[^ ^87^ ^]^
	Te	9.01	1.97	5.49	3.52	^[^ ^87^ ^]^

The chemisorption capture route is based on evidence from the formation of metal‐iodide complexes with chalcogel constituents following the capture of I_2(g)_. Previous studies^[^ ^6^, ^68^, ^69^, ^82^ ^]^ revealed Sn‐based and Sb‐based chalcogels from iodide species including SnI_4_, SnI_4_(S_8_)_2_, and SbI_3_, which were easily detectable with PXRD. The formation of these iodide species allows for a better understanding of how the iodine is interacting with these types of chalcogels.

**Figure 8:**
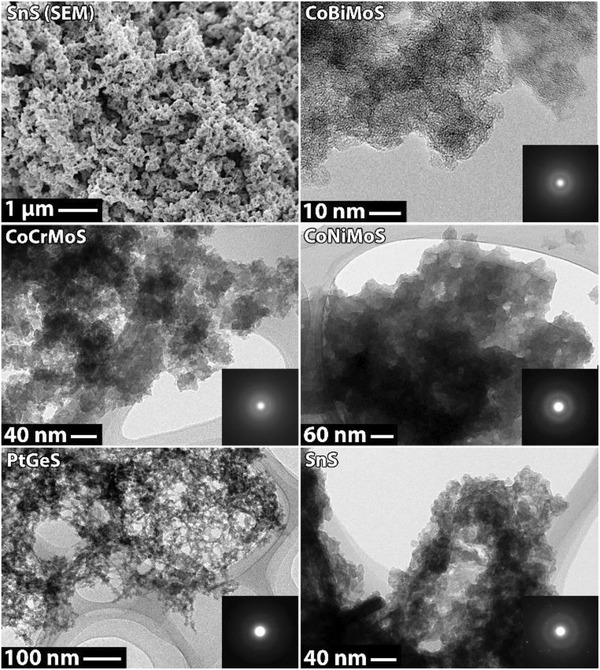
(Top left) SEM and (others) TEM micrographs (with selected area diffraction patterns) for Sn_2_S_3_, Co_0.7_Bi_0.3_MoS_4_, Co_0.7_Cr_0.3_MoS_4_, Co_0.5_Ni_0.5_MoS_4_, and PtGe_2_S_5_ chalcogels. Reproduced with permission.^[^ ^6^ ^]^ Copyright 2013, American Chemical Society.

Chalcogels have very porous networks and appear very similar to oxide‐based counterparts when observed with SEM and TEM (see **Figure** **8**). In some cases, crystalline diffraction has been observed with selected area diffraction (SAD), and this is presumed to be surface oxidation of the materials.

### 4.1 Chalcogels for Iodine Capture

To date, a wide range of chalcogel chemistries have been evaluated for the capture of I_2(g)_ in dilute and/or concentrated streams. Compositions that have been evaluated using concentrated I_2(g)_ streams in a vacuum desiccator at room temperature include PtGe_2_S_5_,^[^ ^15^ ^]^ Sn_2_S_3_,^[^ ^6^, ^68^, ^69^ ^]^ CoNiMo_2_S_8_,^[^ ^6^ ^]^ NiMoS_4_,^[^ ^82^ ^]^ CoMoS_4_,^[^ ^82^ ^]^ Sb_4_Sn_3_S_12_,^[^ ^82^ ^]^ Zn_2_Sn_2_S_6_,^[^ ^82^ ^]^ and KCoS*_x_*.^[^ ^82^ ^]^ For consistency with other sorbents discussed herein, the $m_{\text{I}}\;m_{s}^{-1}$ loading capacities for iodine are provided in **Table** **5** for these chemistries.^[^ ^15^, ^68^, ^82^ ^]^

**Table 5:** Summary of chalcogel chemistries evaluated for I_2(g)_ uptake in saturated environment showing the gel chemistry (sorted alphabetically), the sample identification (ID) from the original work, the measured *SSA* value for that chalcogel, the *SSA*_eq_ value [see Equation (1)], the I_2(g)_ loading based on mass uptake within the vacuum desiccator, and the corresponding reference for the original work

Chalcogel	Sample ID	*SSA* [m^2^ g^−1^]	*SSA*_eq_ [m^2^ g^−1^]	I_2(g)_ loading [$m_{\text{I}}\;m_{s}^{-1}$]	Ref.
CoMoS_4_	CoMoS	360	848	2.00	^[^ ^82^ ^]^
KCoS*_x_*	KCoS	350	–	1.60	^[^ ^82^ ^]^
NiMoS_4_	NiMoS	490	1154	2.25	^[^ ^82^ ^]^
PtGe_2_S_5_	Cg‐5C	360	1200	2.39	^[^ ^15^ ^]^
Sb_4_Sn_3_S_12_	SbSnS	240	817	2.00	^[^ ^82^ ^]^
Sn_2_S_3_	SnS_g_+I	270	999	2.13	^[^ ^68^ ^]^
Zn_2_Sn_2_S_6_	ZnSnS	400	1244	2.25	^[^ ^82^ ^]^

**Figure 9:**
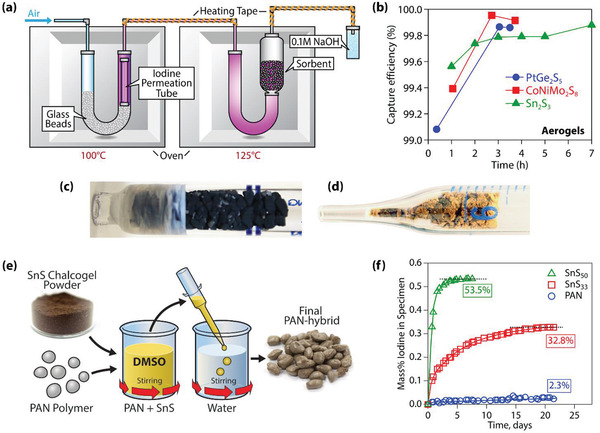
a) Experimental setup for 4.2 ppm iodine capture experiments using DYNACAL permeation tube, b) a summary of the capture as a function of time for PtGe_2_S_5_, CoNiMo_2_S_8_, and Sn_2_S_3_,^[^ ^6^, ^15^ ^]^ c) a close‐up picture of the CoNiMo_2_S_8_ chalcogel after the uptake test, and d) a close‐up picture of the PtGe_2_S_5_ chalcogel after the uptake test; Adapted with permission.^[^ ^6^ ^]^ Copyright 2013, American Chemical Society. e) Schematic showing chalcogel‐PAN composite synthesis process and f) iodine loading as a function of time for two composites and the pure PAN (no chalcogel added); Reproduced with permission.^[^ ^68^ ^]^ Copyright 2014, American Chemical Society.

Additionally, iodine capture was evaluated using a dilute I_2(g)_ stream environment of 4.2 ppm (by volume) in air as the carrier gas. These experiments were conducted using a DYNACAL iodine permeation tube with a permeation rate of 22.8597 ng s^−1^ at 100 °C. This stream was connected to a separate furnace at 150 °C through a heated transfer line where chalcogel sorbents resided; the vent line for this system was placed in 0.1 m NaOH solution that was replaced periodically; this setup can be seen in **Figure** **9**a. Iodine capture efficiency was determined by comparing the iodine breakthrough into the NaOH solution as a function of time, with the sorbent in the column, and those values were compared to the iodine concentration when the sample was not present. Solutions were analyzed using inductively coupled plasma mass spectrometry (ICP‐MS) to determine the iodine concentration. The compositions evaluated using this setup include PtGe_2_S_5_,^[^ ^6^, ^15^ ^]^ CoNiMo_2_S_8_ (see Figure 9c),^[^ ^6^ ^]^ and Sn_2_S_3_ (see Figure 9d).^[^ ^6^ ^]^ In all cases, the uptake over the test duration was > 99 mass% with Sn_2_S_3_ showing the highest uptake over the full test duration demonstrated at >99.5% and increasing with time (see Figure 9b). In a separate study by Subrahmanyam et al.^[^ ^91^ ^]^ (NH_4_)_2_MoS_4_ chalcogels were also demonstrated to have a high affinity for iodine vapors.

One way to increase the mechanical integrity of a sorbent is to embed the active sorbent in a passive matrix (e.g., a polymer‐based scaffold) to create a composite material. Here, the passive matrix essentially dilutes the capacity of the active sorbent on a per‐mass basis since a part of the composite is made up of a passive matrix that either does not interact with the contaminant(s) of interest or only interacts slightly. In a previous study,^[^ ^68^ ^]^ this approach was used with Sn_2_S_3_ chalcogel powders to make chalcogel‐polyacrylonitrile (PAN) composites for iodine capture (Figure 9e). Some benefits from this approach are that the PAN matrix is very porous, stable under elevated temperatures, mechanically robust, and very lightweight, adding minimal mass to the active chalcogel sorbent. In this study, Sn_2_S_3_‐PAN composites were made containing 30, 50, and 70 mass% chalcogel in the PAN. The iodine capacity scaled quite linearly with the amount of Sn_2_S_3_ present in the composite,^[^ ^92^ ^]^ and the loading rates changed with different Sn_2_S_3_ loadings (Figure 9f). These results demonstrate both the passive nature of the PAN matrix and the need for PAN minimization to maximize the iodine capacity in the final composite sorbent.

### 4.2 Chalcogels for the Capture of Uranium and Technetium

Studies were done to evaluate the uptake of uranium‐238 (i.e., ^238^UO_2_^2+^) and technetium‐99 (i.e., ^99^TcO_4_^−^) ions from solution using the following chalcogel chemistries: Co_0.7_Bi_0.3_MoS_4_ (CoBiMoS), Co_0.7_Cr_0.3_MoS_4_ (CoCrMoS), Co_0.5_Ni_0.5_MoS_4_ (CoNiMoS), PtGe_2_S_5_ (PtGeS), and Sn_2_S_3_ (SnS).^[^ ^6^ ^]^ For these experiments, ≈0.05–0.1 g of each chalcogel were loaded into separate 20 mL glass vials with 9 mL DIW; a blank vial was also loaded with 9 mL of DIW. This was followed by the addition of 1 mL solution containing either 10^−5^ m ^99^TcO_4_^−^ added as NaTcO_4_ or 10^−5^ m ^238^UO_2_^2+^ added as UO_2_(NO_3_)_2_ for final concentrations of 10^−6^ m for each target radionuclide. Sample vials were sealed and mixed end‐over‐end for 7 d. Then the supernatant was collected through a syringe with a 0.45 µm filter, and the analyte concentrations were analyzed with ICP‐MS. Capture efficiencies and *K*_d_ values were measured using Equations (3) and (5) (see Section 3), respectively, where *m* is the mass of sorbent material (*V*/*m* ≈ 1000 mL g^−1^) and other variables were defined in Section 3.

(5) $$\text{Efficiency}\;=\;100*\frac{C_{\text{i}}-\;\;C_{\text{t}}}{C_{\text{i}}}$$

The efficiency data and *K*_d_ values from these tests are shown in **Table** **6**. The values revealed a wide range of values for the different chalcogel chemistries where CoBiMoS, PtGeS, and SnS worked well for both ^99^TcO_4_^−^ and ^238^UO_2_^2+^ at ≥87.3% fractional uptake and *K*_d_ values of ≥1.5 × 10^3^. The highest values were observed for PtGeS for both species with *K*_d_s of 3.6 × 10^4^ for ^99^TcO_4_^−^ and 9.4 × 10^4^ for ^238^UO_2_^2+^ along with efficiencies of 98.0% and 99.4%, respectively. The lowest uptake values were found with CoCrMoS at 57.3% and 68.1% efficiencies for ^99^TcO_4_^−^ and ^238^UO_2_^2+^, respectively. These data provide a demonstration that chalcogels could prove useful for the remediation of these radionuclides from aqueous solutions.

**Table 6:** Summary of solution uptake of ^99^TcO_4_^−^ and ^238^UO_2_^2+^ with chalcogels including the efficiency (Eff. %); [see Equation (3)] and the *K*_d_ values [see Equation (4)]. Reproduced with permission.^[^ ^6^ ^]^ Copyright 2013, American Chemical Society

Sample ID	^99^Tc uptake				^238^U uptake			
	Eff. [%]	*K*_d_ [mL g^−1^]	Final pH	Color	Eff. [%]	*K*_d_ [mL g^−1^]	Final pH	Color
CoBiMoS	94.0	1.68 × 10^3^	2.31	Very blue	94.9	1.81 × 10^3^	2.26	Faintly blue
CoCrMoS	57.3	2.88 × 10^2^	2.78	Faintly pink	68.1	3.15 × 10^2^	2.72	Barely pinkish
CoNiMoS	62.2	1.62 × 10^2^	4.12	Pale blue	88.2	8.08 × 10^2^	9.06	Pale blue
PtGeS	98.0	3.61 × 10^4^	4.80	Faintly orange	99.4	9.43 × 10^4^	4.44	Faintly orange
SnS	87.3	1.49 × 10^3^	5.47	Faint yellow	99.1	2.31 × 10^4^	5.53	Faintly yellow
Blank	–	–	5.59	Clear	–	–	5.18	Clear

### 4.3 Chalcogels for the Capture of Heavy Metals

Recent work showed promise for chalcogels for heavy metal remediation; many of these heavy metals could be considered as byproducts to radiological processes and, thus are included in the current review. In a study by Subrahmanyam et al.^[^ ^91^ ^]^ (NH_4_)_2_MoS_4_ chalcogels were demonstrated to have a high affinity for Hg vapors, forming crystalline HgS upon capture with a final composition of Hg_1.9_MoS_4_ after complete exchange of the NH_4_ with Hg. In a study by Pala and Brock,^[^ ^93^ ^]^ ZnS chalcogels were used to remove Pb^2+^ and Hg^2+^ from aqueous solutions. Oh et al.^[^ ^59^ ^]^ showed that Zn_2_Sn*_x_*S_2_ *_x_*_+2_ (*x* = 1, 2, 4) chalcogels can be used to remove Hg^2+^, Pb^2+^, Cd^2+^, Cu^2+^, Zn^2+^, and Fe^2+^ from aqueous solutions with very high *K*_d_ values ranging from 7 × 10^3^ (Fe^2+^) to 2.8 × 10^7^ (Hg^2+^).

### 4.4 Chalcogels for the Capture of Noble Gases

Noble gases are byproducts of nuclear fission, namely, Xe and Kr. Under 40 CFR 190,^[^ ^94^ ^]^ the U.S. Environmental Protection Agency requires the capture ^85^Kr evolved from a nuclear fuel reprocessing facility, if one were to be built in the United States. The concentration of Xe present within irradiated nuclear fuel is nominally 10× the total Kr concentration and this combined with the unreactive nature of noble bases makes the selective capture of Kr even more challenging. The traditional option for capturing noble gases has been cryogenic distillation,^[^ ^95^, ^96^, ^97^, ^98^, ^99^ ^]^ but new MOF sorbents^[^ ^100^, ^101^, ^102^ ^]^ have shown promise as alternatives to distillation.

**Figure 10:**
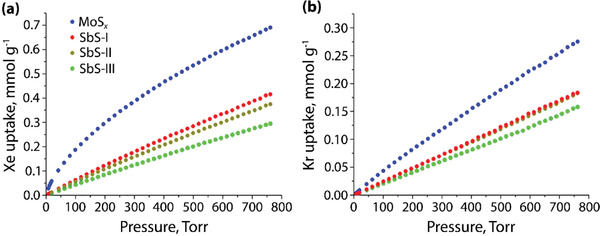
a) Xe and b) Kr adsorption isotherms recorded at 273 K up to 1 bar. Adapted with permission.^[^ ^103^ ^]^ Copyright 2017, American Chemical Society.

A paper by Subrahmanyam et al.^[^ ^103^ ^]^ revealed that chalcogels could be used to capture Xe from the noble gas mixture. Here, four different chalcogels were utilized including (NH_4_)_0.03_MoS_4_ (MoS*_x_*), Na_0.3_Sb_2_S_3_ (SbS‐I), K_0.15_Na_0.3_Sb_2_S_2.5_ (SbS‐II), and Na_0.1_Sb_2_S_3_ (SbS‐III) with *SSA* values of 128–290 m^2^ g^−1^. The MoS*_x_* chalcogel showed the highest uptake for both Xe and Kr as shown in **Figure** **10**a,b, respectively. Calculations were conducted using the ideal adsorbed solution theory (IAST) to look at Xe/Kr selectivities among the four chalcogels and the MoS*_x_* chalcogel exhibited the highest value of 6.0 compared to selectivities of 2.0–2.8 for all Sb‐S chalcogels. It is hypothesized that the differences in selectivities between the Mo‐S and Sb‐S chalcogels are possibly due to the differences in metal ion oxidation state (i.e., Mo^4+^ vs Sb^3+^) as well as crystal radii.

## 5 Heavy Metal Capture with Carbon‐Based Aerogel Composites

For removal of heavy radionuclides from aqueous solutions, different carbon‐based functionalized aerogel composites have been developed to improve adsorption capacities while maintaining high porosities and high *SSA* values while also allowing for the incorporation of various getters in the structures.^[^ ^104^, ^105^, ^106^, ^107^, ^108^, ^109^, ^110^ ^]^ **Table** **7** shows the adsorption capacities of radionuclides by various sorbents in the literature;^[^ ^105^, ^106^, ^107^, ^108^ ^]^ also included in Table 7 are additional data for similar compounds without aerogels included for comparison.^[^ ^111^, ^112^ ^]^

**Table 7:** Comparison of the adsorption capacities of various sorbents on radioactive and surrogate ions

Type	Sorbents	Target species	Species capacity [mg g^−1^]	Solution pH	Temperature [K]	Ref.
With aerogels	Graphene aerogel	U	131	5.5	298	^[^ ^105^ ^]^
	PANI‐graphene aerogel	U	242	5.5	298	^[^ ^105^ ^]^
	Fe^0^‐PANI‐graphene aerogel	U	350	5.5	298	^[^ ^105^ ^]^
	Fungal hypha‐graphene oxide aerogel	U	288	5	293	^[^ ^106^ ^]^
	Konjac glucomannan/graphene oxide aerogel	U	513	5	313	^[^ ^107^ ^]^
	SiO_2_‐ZrO_2_‐calcium alginate aerogels	Ce	232	7	308	^[^ ^108^ ^]^
	Graphene oxide/polypyrrole	U	147	5	298	^[^ ^117^ ^]^
	Na_2_Ti_3_O_7_‐carbon aerogel	Rb	94.9	5.5	298	^[^ ^110^ ^]^
	Na_2_Ti_3_O_7_‐carbon aerogel	Cs	193	5.5	298	^[^ ^110^ ^]^
Without aerogels	Carbonaceous nanofiber	U	125	4.5	298	^[^ ^111^ ^]^
	Carbonaceous nanofiber	Eu	91	4.5	298	^[^ ^111^ ^]^
	Graphene oxide	U	56	3.8	298	^[^ ^112^ ^]^
	Graphene oxide/α‐MnO_2_	U	185	3.8	298	^[^ ^112^ ^]^
	Graphene oxide	Th	154	3.8	298	^[^ ^112^ ^]^
	Graphene oxide/α‐MnO_2_	Th	498	3.8	298	^[^ ^112^ ^]^

Coleman et al.^[^ ^104^ ^]^ developed a granulated activated carbon (GAC)‐silica aerogel composite for uranium removal from aqueous solution. Hydrophobic GAC‐aerogel composites were synthesized and modified with phosphoric acid, phosphonate, or Ca(NO_3_)_2_, and their uranium adsorption capacities were evaluated in 100 ppb uranium and pH 7 surrogate solution.^[^ ^104^ ^]^ The results showed that GAC‐aerogel composites were better adsorbents compared to normal GAC.

Chen et al.^[^ ^105^ ^]^ developed a Fe^0^‐polyaniline‐graphene aerogel composite (Fe^0^‐PANI‐GA) for U^6+^ removal from acidic solutions. Graphene aerogel is composed of cross‐linked graphene sheets with robust porous structures that can incorporate getters and has been studied for removal of heavy metal ions.^[^ ^113^ ^]^ Zero‐valent iron (Fe^0^) is an effective adsorbent for heavy metal ions due to more reactive sites on the surface,^[^ ^114^, ^115^ ^]^ and polyaniline contains abundant primary and secondary amino groups that help assist the adsorption of heavy metal ions.^[^ ^116^ ^]^ The Fe^0^‐PANI‐GA composite was more effective for removal of U^6+^ compared to GA or PANI‐GA alone in acidic solution, and the maximum sorption capacity was 350 mg g^−1^ in pH 5 solution at room temperature (see Table 7).^[^ ^105^ ^]^

Liu et al.^[^ ^110^ ^]^ developed Na_2_Ti_3_O_7_‐carbon aerogel for removal of Rb^+^ and Cs^+^ cations from aqueous solution. The adsorption mechanism involves replacement of Na^+^ with Rb^+^ and Cs^+^ cations in Na_2_Ti_3_O_7_ and complexation of hydroxyl functional groups from Na_2_Ti_3_O_7_‐carbon aerogel with Rb^+^ and Cs^+^ cations.^[^ ^110^ ^]^ The maximum adsorption capacity of Na_2_Ti_3_O_7_‐carbon aerogel for Rb^+^ and Cs^+^ cations were 1.11 mmol g^−1^ (94.9 mg g^−1^) and 1.45 mmol g^−1^ (193 mg g^−1^), respectively, from a solution of pH 5.5.^[^ ^110^ ^]^

Li et al.^[^ ^106^ ^]^ used a combination of microorganisms and aerogels to develop an adsorbent of fungal hypha‐graphene oxide aerogel composite for uranium removal from aqueous solutions. Fungal hypha contain a large amount of phosphonate, hydroxyl, and amine groups on the cell wall that have strong chelation with uranium ions.^[^ ^106^ ^]^ The sorption capacity of fungal hypha‐graphene oxide aerogel composites for U^6+^ ions in a solution of pH 5 was 288 mg g^−1^.^[^ ^106^ ^]^

Chen et al.^[^ ^107^ ^]^ developed konjac glucomannan/graphene oxide composite aerogel for U^6+^ removal from simulated wastewater. Konjac glucomannan is a renewable natural resource that can adsorb radionuclides, but with a relatively low adsorption capacity. By combining with graphene oxide and aerogel, its absorption capacity can be increased significantly.^[^ ^107^ ^]^ The maximum adsorption capacity of U^6+^ from pH 5 solution at 313 K was 513 mg g^−1^.^[^ ^107^ ^]^

Zhao et al.^[^ ^108^ ^]^ removed cerium, a surrogate of plutonium and minor actinides, from an aqueous solution using SiO_2_‐ZrO_2_‐calcium alginate aerogels and showed that Ca and Zr sites were occupied by Ce^4+^ cations with the maximum adsorption capacity of 232 mg g^−1^. Calcium alginate is a natural biological polymer that can be extracted from brown algae.

Zhu et al.^[^ ^109^ ^]^ demonstrated that low cost and non‐toxic cotton‐carbon aerogel with high cellulose content can be used as an adsorbent for removal of Sr^2+^ cations from pH 5–7 solutions. The maximum removal efficiency of cotton‐carbon aerogel for Sr^2+^ removal was 60.2% from a solution of pH 6, and cotton‐carbon aerogel was reusable for five adsorption and desorption cycles.^[^ ^109^ ^]^

## 6 Summary and Conclusions

This progress report provides a review of recent work in the area of functional aerogels and xerogels for environmental remediation applications. The materials discussed herein include sorbents for liquid and/or gaseous streams. Sorbents discussed include metal‐functionalized silica‐based aerogels and xerogels, sulfide‐based aerogels, and carbon‐containing aerogel composites for target species such as Cd^2+^, Ce^4+^, Cs^+^, Cu^2+^, Fe^2+^, Hg^2+^, Hg_(g)_, I^−^, IO_3_^−^, Kr, Pb^2+^, Rb^+^, Sr^2+^, ^99^Tc^7+^, U^6+^, Xe, and Zn^2+^. These sorbents have a range of specific surface areas, porosities, and capacities for these contaminants.

Depending on the application, different materials are more suitable than others. For instance, some aerogels are quite fragile and friable; these might not be ideal for applications requiring high‐flow gas streams or extreme‐pH aqueous environments. For such environments, additional techniques can be implemented such as embedding the active sorbent in a passive matrix, as was done with the chalcogel‐PAN composites; this added mass of the inactive matrix will decrease the capacity of the overall composite, but with the added benefit of increased mechanical integrity.

Overall, oxide‐based aerogels and xerogels are likely less expensive to make than chalcogels, due to the commercially availability of a wide range of alkoxide precursors and a very limited availability of chalcogel precursors; additionally, commercially available chalcogel precursors also tend to be rather expensive. The added benefit of chalcogels, however, is that they are oftentimes inherently “functionalized” for these types of applications where the as‐made sorbents can be used to capture species of interest. In contrast, oxide‐based gels often require the addition of a getter species, e.g., Ag^+^/Ag^0^.

The primary conclusion of this work is that plenty of open spaces exist on this topic in the literature and new applications are being discovered regularly. Additional topics of interest and challenges in this area are plentiful, some of which are described below. For instance, future work should focus on finding non‐hazardous metal getters. Silver is a hazardous material as well as a precious metal. With recent work showing promise for other getters such as Cu, Sn, and Sb, this provides an opportunity for cheaper and less hazardous sorbents. A limitation with aerogel production is the requirement for critical point drying using autoclaves and supercritical fluids. This can be avoided if xerogels are pursued instead. Plus, xerogels can be made with minimal *SSA* loss, as was discussed herein, and they tend to have significantly higher mechanical stability than the aerogel counterparts. A large‐scale production process for making xerogels would greatly improve the workflow.

Very little work has been done looking at gel‐polymer composites for these applications and this type of approach could be used to further push the limits of environmental remediation applications, e.g., under conditions where gels (without added support) might not fare well from a mechanical integrity perspective. The added disadvantage of the polymer being part of the composite is that the sorbent cannot be easily consolidated into a waste form if the polymer remains, unless it is selectively removed (e.g., by burning it off) beforehand. However, the composite sorbent could be loaded into a secondary matrix for disposal, such as a cement or grout.

This leads into the final point, which is that the fate of the contaminant‐loaded sorbent need be considered. Very little has been done to attempt consolidation of contaminant‐loaded gels into a waste form suitable for long‐term disposal. This remains an area that is wide open for future research. Since these sorbents are porous, at a minimum, they could be hot pressed into a dense monolithic waste form. However, it would be preferred if the contaminants could be removed and the base sorbent recycled for future use.

## Conflict of Interest

The authors declare no conflict of interest.

## Supporting information

Supporting Information
