# Supplementary material for: Environmental Remediation with Functional Aerogels and Xerogels
Source: Glob Chall. 2020 Jun 17;4(10):2000013. doi: 10.1002/gch2.202000013 (PMC7533867; doi:10.1002/gch2.202000013)
Supplement: Supplementary file 1 — Supporting Information [file GCH2-4-2000013-s001.pdf]

Copyright WILEY-VCH Verlag GmbH & Co. KGaA, 69469 Weinheim, Germany, 2020.

Supporting Information

for *Global Challenges,* DOI: 10.1002/gch2.202000013

Environmental Remediation with Functional Aerogels and Xerogels

Brian J. Riley and Saehwa Chong*

Supporting Information

Radionuclide remediation with functional aerogels and xerogels

Brian J. Riley and Saehwa Chong,*

Dr. B. J. Riley, Dr. S. Chong
Pacific Northwest National Laboratory, 902 Battelle Blvd, Richland, WA, 99352, USA

E-mail: brian.riley@pnnl.gov

Copyright WILEY-VCH Verlag GmbH & Co. KGaA, 69469 Weinheim, Germany, 2018.

Supporting Information

Functional aerogel scaffolds for the capture and immobilization of radionuclides

Brian J. Riley, Saehwa Chong*

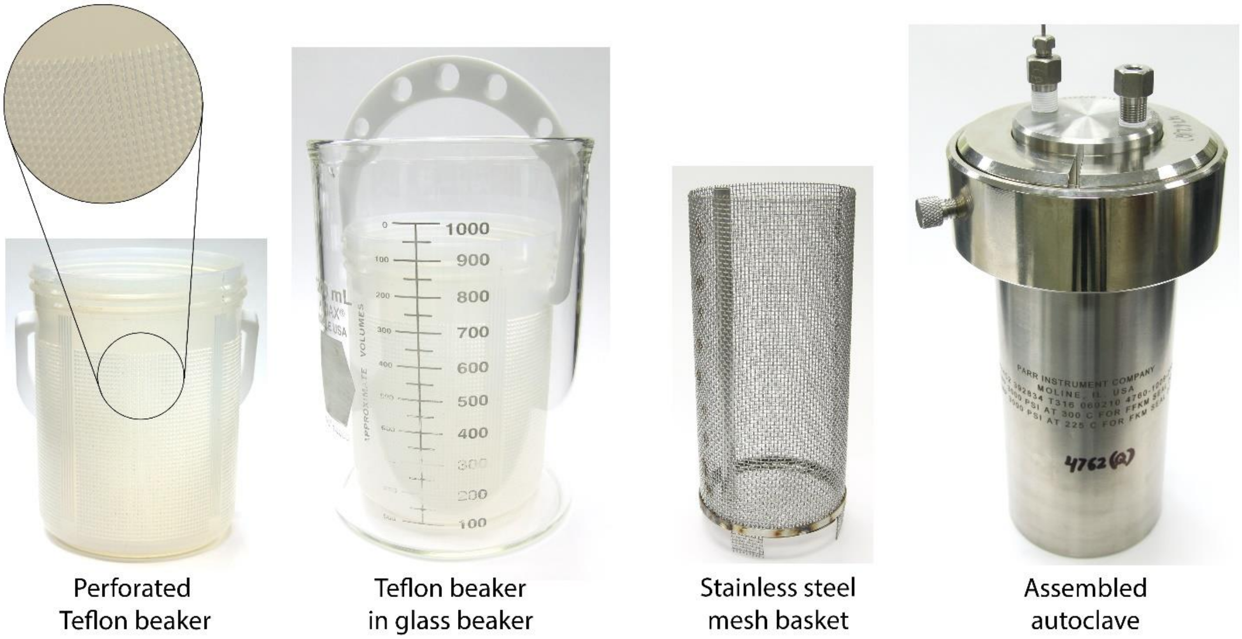

Figure S1. Tools for preparing gels during solvent exchange and critical point drying including a perforated Teflon beaker with a handle for quick and easy solvent exchanging that fits into a 1-L beaker as well as a stainless-steel mesh basket that can be used to hold gels in an autoclave [e.g., Parr Instruments 4762(Q)] during critical point drying.

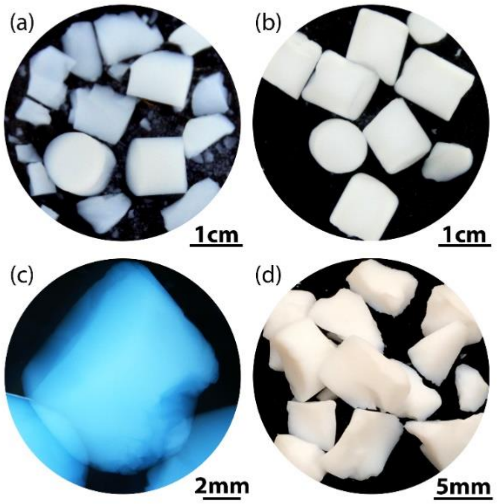

Figure S2. Pictures of (a) Na-Al-Si-O alcogels, (b) Na-Al-Si-O aerogels, (c) Al-Si-O alcogels, and (d) Al-Si-O aerogels from Riley et al.[S1] This figure was reprinted with permission. ©2017, American Chemical Society.

References

[S1] B.J. Riley, J.O. Kroll, J.A. Peterson, J. Matyáš, M.J. Olszta, X. Li, J.D. Vienna, *Environ. Sci. Technol.*, **2017**, *9*, 32907.
